# Satisfaction with telepsychiatry and mental health stigma among health sciences students in Egyptian universities

**DOI:** 10.1038/s41598-026-60516-8

**Published:** 2026-07-10

**Authors:** Mohammed. N. Abdelaziz, Ranim. A. Karaman, Hajer Azzam, Mariam. M. Elganainy, Roaa. M. Ebrahim, Dalia.S.Basmsam, Mohammed.A. Elnour, Doha. S. Hodhod, TSRA collaborators, Yomna.M. Elhosary, Abdelrahman Fareed, Nourhan.G. Mady, Hiba Khalid Mohammed Alzubair, Muhammad Emad Al-Qabbari, Omnia Yousry Elhadidy, Abdelrahman Abdelhakim Assaf

**Affiliations:** 1 Faculty of Medicine, Mansoura University, Mansoura, Egypt; 2 Faculty of Medicine, Tanta University, Tanta, Egypt; 3 Faculty of Medicine, Alexandria University, Alexandria, Egypt; 4 Faculty of Medicine, October 6 University, Giza, Egypt; 5 Psychiatry Department, Faculty of Medicine, Mansoura University, Mansoura, Egypt; 6 Neuropsychiatry Department, Faculty of Medicine, Tanta University, Tanta, Egypt

**Keywords:** Telepsychiatry, Mental health stigma, Satisfaction, Behavioral health, Diseases, Health care, Medical research, Psychology, Psychology

## Abstract

Health sciences students have a higher incidence of mental health disorders; however, stigma and barriers have complicated help-seeking. Telepsychiatry may be a solution, but acceptance, satisfaction, and stigma in this population, especially in Egypt, remain unclear. This study aimed to evaluate the levels of satisfaction regarding telepsychiatry, stigma perceived with respect to mental illness, among health sciences students towards mental health services. It also aims to assess sociodemographic factors associated with these outcomes. This was a questionnaire-based cross-sectional study conducted among 799 students from various universities in Egypt. It was conducted between August and November 2025, and the questionnaire involved sociodemographic data, the telepsychiatry satisfaction scale (21 items), and the STIG-9 stigma scale. Data were analyzed using descriptive statistics, chi-square and t-tests, and linear regression, with significance set at p < 0.05. Overall, participants had a mean age of 21.4 years, while 66.8% of participants were female. In total, 24.0% agreed, and 18.4% strongly agreed to being satisfied with telepsychiatry, with a mean total satisfaction score of 67.84/105. The mean STIG‑9 stigma score was 14.44/27. Linear regression identified sociodemographic variables independently associated with satisfaction and stigma; higher stigma was also positively associated with higher satisfaction. In this cohort of Egyptian health sciences students with prior telepsychiatry experience, satisfaction with telepsychiatry was positively associated with perceived stigma. These findings suggest that addressing socio-cultural stigma may support the acceptability of telepsychiatry and improve engagement with digital mental health services in similar student populations.

**Supplementary Information:**

The online version contains supplementary material available at 10.1038/s41598-026-60516-8.

## Introduction

Mental health disorders constitute a foremost public health challenge worldwide, affecting 1 billion people according to the World Health Organization^1^. This burden disproportionately impacts young adults, particularly university students, whose psychological stressors—including academic demands, social pressures, and developmental transitions—exacerbate adjustment difficulties^1,2^. Health sciences students emerge as especially vulnerable, facing rigorous academic demands, financial strains, exposure to illness and death during clinical training, and preparation for one of medicine’s most demanding professions^3,4^. Prevalence data underscore this crisis: a systematic review and meta-analysis during the COVID-19 pandemic reported pooled rates of 45% anxiety and 48% depression among health sciences students^2^, while Egyptian studies confirm similar patterns, including 42.9% depression et al.-Azhar University^5^ and nearly 59% of students with at least one mental disorder at Mansoura Faculty of Medicine^6^. These findings highlight an urgent need for accessible, tailored mental health services. Treatment gaps amplify this burden, particularly in low- and middle-income countries^7^. In the Middle East and North Africa, cultural stigma—viewing mental illness as personal weakness, spiritual failing, or family shame—deters help-seeking among youth and health sciences students, compounded by norms emphasizing family privacy and self-reliance^8,9^.

Telepsychiatry, the delivery of psychiatric services via telecommunications such as videoconferencing and telephone consultations, offers a promising solution by mitigating geographical, transportation, and stigma-related barriers through private, home-based care^10,11^. Evidence demonstrates its equivalence to in-person care across psychiatric conditions, with high satisfaction among patients and providers^10^. The COVID-19 pandemic accelerated its global adoption amid social distancing and infection risks, transitioning mental health services to virtual platforms; approximately 82% of patients rated their telepsychiatry experience as excellent or good^12,13^. Optimizing services for this high-risk group thus requires assessing their satisfaction with telepsychiatry^14^.

Stigma further impedes utilization, encompassing societal perceptions of mental illness^15^. The Stigma-9 Questionnaire (STIG-9) measures its cognitive, behavioral, and affective dimensions, with higher scores signaling anticipated negative views^16^. In Egypt—a developing Middle Eastern and North African nation with limited mental health system capacity, few trained professionals, and entrenched negative societal perceptions toward mental health seeking^17^—telepsychiatry implementation faces distinct hurdles. Nevertheless, rising smartphone penetration and digital literacy among the youth create conducive circumstances for telemedicine expansion. Health sciences students in Egypt represent an ideal population to study due to their technological literacy and heavy mental health burdens^18–20^. This study aimed to^1^ assess telepsychiatry satisfaction among Egyptian university health sciences students using a validated 21-item scale^2^; measure perceived stigma via STIG-9; and^3^ identify sociodemographic variables of satisfaction and stigma.

## Methods

### Population and study design

**Fig. 1:**
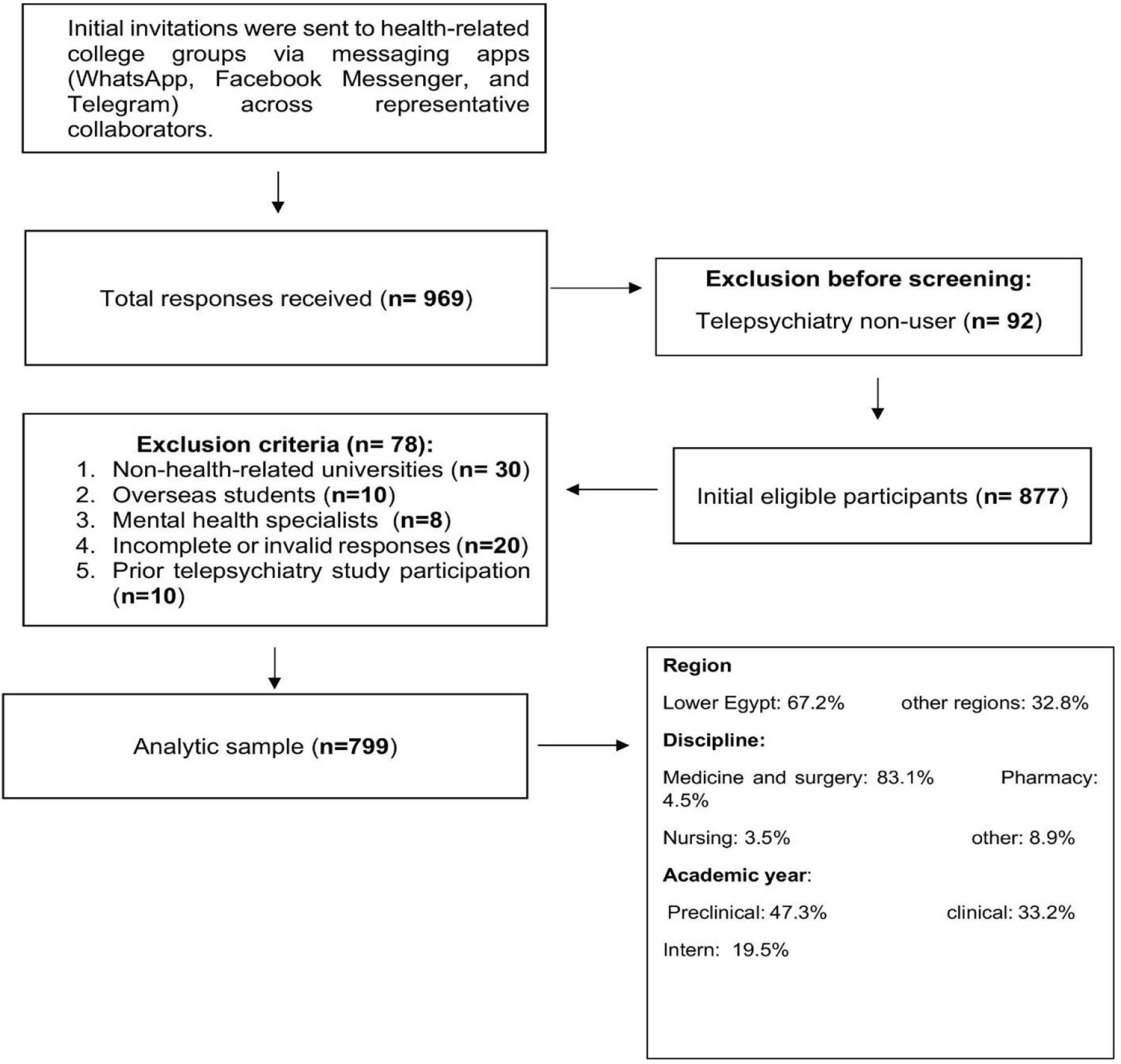
Flow chart diagram showing recruitment sources, exclusions, and final analytic sample by key strata (region, discipline, year).

This cross-sectional, questionnaire-based study was conducted among Egyptian university students from August 2025 to November 2025. The study specifically targeted health sciences students currently enrolled in accredited public or private Egyptian universities. To ensure the validity of the satisfaction metrics, the study implemented a rigorous participant selection process. Inclusion in the analytic sample was based on self-reported prior use of telepsychiatry services. Eligible participants were required to be 18 years or older and recruited across all academic stages (preclinical, clinical, and internship). Participants were required to possess internet-enabled digital devices (smartphones, tablets, or computers) to access the survey and provide electronic informed consent. Exclusion criteria included students in non-health-related faculties, those studying medicine abroad, or individuals with specialized mental health training (e.g., certified counselors) to ensure the sample reflected the general undergraduate health sciences population. Furthermore, students who had previously participated in formal telepsychiatry research were excluded to minimize prior-exposure bias. Participant flow, including recruitment sources and the final analytic sample, is illustrated in Fig. 1.

The sample size was calculated using OpenEpi (https://www.openepi.com/SampleSize/SSPropor.htm). Assuming a satisfaction level of 43% according to a previous study^21^ (p = 0.43), with a 5% margin of error and 95% confidence interval. Considering the large population of health-related students in Egypt, the population was treated as infinite. Thus, the minimum required sample size is 565. Considering 20% non-response rate encountered in online survey-based research among healthcare students and professionals, the required sample size was inflated to 706. Such an adjustment aligns with common practices in previous telemedicine perception and satisfaction studies targeting medical and health-related students in the Middle East^22^.

The study used a convenience sampling approach. Survey administered using Google Forms (Google Inc.) across all sites. Single dataset collected via a centralized Google Forms link distributed by collaborators affiliated with Egyptian universities. Collaborators shared the survey link through student-oriented communication channels, including academic and social media groups commonly used by health sciences students. Participation was voluntary, and no monetary, academic, or other incentives were provided. Before participation, students were informed about the study aims, confidentiality, and anonymity, and electronic informed consent was obtained. Although this approach enabled broad and efficient recruitment, it may have introduced selection bias by preferentially reaching students who were more digitally active or more interested in mental health-related topics. This limitation was acknowledged when interpreting the findings. Several attempts were made to minimize bias by ensuring that information regarding the purpose of the survey was communicated well, along with reminders for participation. After completing all questions, participants were later instructed to submit the web form to the web server. All responses are automatically aggregated into one dataset and exported as Excel for cleaning/analysis. No multiple datasets or merging required. To ensure data integrity, duplicate entries were minimized using both preventative platform restrictions and post-hoc data cleaning procedures. During the survey administration stage, the ‘Limit to 1 response’ feature on Google Forms was activated, restricting submissions to a single entry per Google account. During the data cleaning phase, the exported dataset was manually screened for potential duplicates. Suspected duplicates were judged based on an exact matching matrix of all sociodemographic variables (age, gender, university discipline, academic year, and region) coupled with closely clustered submission timestamps. Any identical overlapping profiles resulting from submission or platform errors were removed prior to final statistical analysis.

### Data collection procedure

The structured questionnaire developed from reviewing the existing literature contained three parts: demographic information, telepsychiatry patient satisfaction and experience questionnaire, and mental health stigma questionnaire. This was then validated by a panel of experts in public health and psychiatry departments. Reliability and internal consistency were evaluated using the Cronbach’s alpha test. The reliability of the questionnaire was piloted on 30 students for final analysis. Scales of satisfaction questionnaires showed acceptable reliability (Cronbach’s a = 0.93). The scales of the STIG-9 questionnaire showed acceptable reliability (Cronbach’s a = 0.85). In this study, the instruments were applied to health sciences students in Egyptian universities and within a telepsychiatry-related context, which may differ in cultural, linguistic, and contextual relevance from the original validation samples. Therefore, reassessment of reliability and contextual validity was considered necessary before use in the current population. The questionnaire was administered in English because English is the primary language of instruction across health sciences programs in Egyptian universities, and students are routinely exposed to English-language educational and clinical materials throughout their academic training. To ensure suitability for the target population, comprehensibility, clarity, and linguistic appropriateness of the questionnaire items were additionally assessed during pilot testing before final administration. Based on pilot feedback, the questionnaire was considered understandable and appropriate for use in the study population. It is four parts: Sect. 1: demographic information: includes seven items collecting baseline characteristics of participants, including age, gender (male/female), academic level (preclinical, clinical, or intern), economic status (high, middle, or low), type of residence (urban or rural), state of residence, and nationality (Egyptian or non-Egyptian). An open-ended question assessed participants’ prior experience with mental health issues. A primary ‘Filter Question’—"Have you ever used telepsychiatry services?"—was utilized to distinguish between users and non-users. Non-telepsychiatry users were excluded during the data cleaning phase. Section 2: The 21-item telepsychiatry satisfaction and experience questionnaire was used exactly as validated by Serhal et al.^23^; full items/domains in (Supplementary material). All participants rated their actual experiences on a 5-point Likert scale (1 = Strongly Disagree, 5 = Strongly Agree). Overall satisfaction was additionally summarized using the single global item (Item 21: ‘Overall, I am satisfied with the Telepsychiatry appointment’), and the proportion of participants selecting ‘agree’ or ‘strongly agree’ was reported. Section 3: Mental Health Stigma features were consistent with the STIG-9 (Stigma-9) questionnaire^19^ with a 4-point pressure scale (disagree, somewhat disagree, somewhat agree, agree) to measure stigma. Items rated on this scale addressed what society views as very negative peer traits, including reduced credibility, perceived danger, hesitancy in business transactions, negative social judgment, attribution of personal weakness, reluctance to care for children, employment discrimination, avoidance of romantic relationships, and neighborhood-based discomfort. Each item of the stigma scale is rated on a Likert-type scale, and item scores were summed to obtain a total stigma score. Higher total scores indicate higher perceived mental health-related stigma. Satisfaction item responses were summed to generate a composite score. Higher scores reflected more positive satisfaction with telepsychiatry services. No weighting was applied to individual items. These composite scores were subsequently used as continuous variables in the statistical analyses. Response rates per university/governorate were not tracked due to decentralized convenience sampling via student representatives.

### Statistical analysis

We used IBM Statistical Package for Social Science (SPSS) software version 25 for data entry and statistical analysis. Categorical data were summarized and described as numbers and percentages. Continuous data were summarized and described as mean and SD, as data were normally distributed according to normality plots. Categorical data were compared using chi-square and Fisher’s exact tests. To compare continuous data, we used the Student’s t-test to compare between two groups and one-way ANOVA to compare between more than two groups. Data normality confirmed via Shapiro–Wilk tests. Pearson correlations were used for bivariate associations between continuous variables. Subsequently, multiple linear regression analyses were performed to identify factors independently associated with the satisfaction score and stigma score. Two separate regression models were constructed, with satisfaction score and stigma score serving as the dependent variables. Selection of independent variables for the multivariable linear regression models employed a hybrid theory-driven and data-driven approach, guided by established biostatistical principles to balance clinical relevance, statistical power, and model parsimony. All eligible variables were then simultaneously entered into the models using forced entry. In addition, stigma score and satisfaction score were included in the respective regression models to examine their potential association. Regression coefficients (B) with their corresponding standard errors (SE), R values, and exact p-values, 95% confidence intervals (95% CI) were reported. Multicollinearity among independent variables was assessed using the variance inflation factor (VIF), with values < 5 indicating no evidence of multicollinearity. Results were considered statistically significant when the p-value was ≤ 0.05. Due to small sample sizes in several geographic subgroups, the region variable was dichotomized into Lower Egypt (Delta) versus other regions for regression analyses to ensure statistical stability and avoid unreliable estimates from sparse categories.

## Results

The sociodemographic characteristics of the study sample are shown in Table 1. Our study included 799 participants, whose mean age was 21.44 (2.2), and the majority were female (66.8%). Most participants study Medicine and Surgery, are pre-clinical students (47.3%), and have a middle economic status (88.7%). The economic status was classified based on income brackets (low: < 10,000 EGP; middle: 10,000–30,000 EGP; high: > 30,000 EGP). Egyptian participants represented the majority (79.1%), from urban residence (57.9%) and Lower Egypt (67.2%). A descriptive comparison across geographic regions was presented in Table S1. Of participants, 37.9% reported that they have experienced mental health issues. Of them, 39.9% reported anxiety disorders and 38.3% reported mood disorders.

**Table 1:** Sociodemographic characteristics of the study population (n = 799).

Parameters	Mean/N	SD/%
Age	21.44	2.2
Gender		
Male	265	33.2%
Female	534	66.8%
Field of study		
Medicine and surgery	664	83.1%
Pharmacy	36	4.5%
Nursing	28	3.5%
Other	71	8.9%
Academic level		
Preclinical	378	47.3%
Clinical	265	33.2%
Intern	156	19.5%
Economic status		
High	55	6.9%
Middle	709	88.7%
Low	35	4.4%
Residence		
Urban	463	57.9%
Rural	336	42.1%
Region		
Lower Egypt (Delta)	537	67.2%
Other regions	262	32.8%
Nationality		
Egyptian	632	79.1%
Non-Egyptian	167	20.9%
Have you ever experienced mental health issues?		
Yes	303	37.9%
No	496	62.1%
If yes, please specify (N = 303)		
Mood disorders (e.g., Depression, Mania, Bipolar)	116	38.3%
Psychotic disorders (e.g., Schizophrenia)	7	2.3%
Anxiety disorders	121	39.9%
Personality disorders	15	5%
Behavioral disorders (e.g., ADHD, Autism)	15	5%
Others	29	9.6%

Table 2 presents the summary of satisfaction with Telepsychiatry. Full distribution of responses to the telepsychiatry satisfaction questionnaire using a 5-point Likert scale was presented in Table S2. Factors assessed by the questionnaire reported the following means: access and timeliness, 12.36 out of 20 (3.6); appropriateness, 9.87 out of 15 (2.85); effectiveness, 19.12 out of 30 (5.2); efficiency, 6.36 out of 10 (1.98); and safety, 16.78 out of 25 (4.87). Overall, 42.4% of participants reported satisfaction with telepsychiatry appointments, defined as those who selected ‘agree’ or ‘strongly agree’ on the global satisfaction item (Item 21). The total score of satisfaction questions had a mean of 67.84 out of 105 (17.9). Table 3 shows the summary of STIG-9 questions. The total score of stigma perceptions showed a mean of 14.44 out of 27 (5.98).

**Table 2:** Satisfaction with Telepsychiatry (n = 799).

Agree N (%)	Strongly agree N (%)	Mean (SD)
Factor 1: Access and timeliness/20		12.36 (3.6)
1. I am satisfied with the length of time I had to wait between my referral and the Telepsychiatry appointment		
94 (11.8%)	100 (12.5%)	
2. It was easy to book my Telepsychiatry appointment		
140 (17.5%)	120 (15%)	
16. I am confident that I will be able to follow the psychiatrist’s recommendations		
206 (25.8%)	141 (17.6%)	
18. The physical location of my Telepsychiatry appointment was convenient for me to get to		
152 (19%)	112 (14%)	
Factor 2: Appropriateness/15		9.87 (2.85)
8. I believe Telepsychiatry is just as effective as an in-person psychiatry appointment		
156 (19.5%)	99 (12.4%)	
11. The psychiatrist understood my concerns		
220 (27.5%)	177 (22.2%)	
14. The psychiatrist involved me in decisions about my treatment plan		
217 (27.2%)	153 (19.1%)	
Factor 3: Effectiveness/30		19.12 (5.2)
3. During my Telepsychiatry appointment, I was able to see the psychiatrist clearly		
153 (19.1%)	150 (18.8%)	
4. During my Telepsychiatry appointment, I was able to hear the psychiatrist clearly		
169 (21.2%)	160 (20%)	
5. I am confident that the psychiatrist and my health care providers are working as a team		
175 (21.9%)	172 (21.5%)	
13. The psychiatrist explained my diagnosis in a way that I could understand		
203 (25.4%)	191 (23.9%)	
19. I experienced a significant improvement in my mental health while I was waiting for my Telepsychiatry appointment		
168 (21%)	113 (14.1%)	
20. I experienced a significant decline in my mental health while I was waiting for my Telepsychiatry appointment		
101 (12.6%)	70 (8.8%)	
Factor 4: Efficiency/10		6.36 (1.98)
6. I feel that there was an adequate amount of time allotted for the Telepsychiatry appointment		
180 (22.5%)	109 (13.6%)	
9. I was able to get an appointment through Telepsychiatry sooner than an in-person psychiatry appointment		
159 (19.9%)	130 (16.3%)	
Factor 5: Safety/25		16.78 (4.87)
7. I felt comfortable during my Telepsychiatry appointment		
178 (22.3%)	149 (18.6%)	
10. I felt that confidentiality was protected throughout my Telepsychiatry appointment		
182 (22.8%)	147 (18.4%)	
12. The psychiatrist treated me with courtesy and respect		
199 (24.9%)	240 (30%)	
15. The psychiatrist explained the benefits and risks of any medications he/she recommended		
196 (24.5%)	154 (19.3%)	
17. I understand what to do if I have a mental health emergency following this appointment		
184 (23%)	150 (18.8%)	
Overall satisfaction		
21. Overall, I am satisfied with the Telepsychiatry appointment		
192 (24%)	147 (18.4%)	
Total score/105		67.84 (17.9)

**Table 3:** Summary of STIG-9 questionnaire (n = 799).

Disagree	Somewhat disagree	Somewhat agree	Agree	Mean (SD)
[I think that most people take the opinion of someone who has been treated for a mental illness less seriously.]				
125 (15.6%)	200 (25%)	346 (43.3%)	128 (16%)	1.6 (0.94)
[I think that most people consider someone who has been treated for a mental illness to be dangerous.]				
133 (16.6%)	240 (30%)	295 (36.9%)	131 (16.4%)	1.53 (0.95)
[I think that most people hesitate to do business with someone who has been treated for a mental illness.]				
113 (14.1%)	209 (26.2%)	339 (42.4%)	138 (17.3%)	1.63 (0.93)
[I think that most people think badly of someone who has been treated for a mental illness]				
103 (12.9%)	215 (26.9%)	322 (40.3%)	159 (19.9%)	1.67 (0.94)
[I think that most people consider mental illness to be a sign of personal weakness]				
116 (14.5%)	189 (23.7%)	321 (40.2%)	173 (21.7%)	1.69 (0.97)
[I think that most people hesitate to entrust their child with]				
91 (11.4%)	204 (25.5%)	335 (41.9%)	169 (21.2%)	1.73 (0.92)
[I think that most people do not even take a look at an application from someone who has been treated for a mental illness]				
150 (18.8%)	230 (28.8%)	326 (40.8%)	93 (11.6%)	1.45 (0.93)
[I think that most people do not enter into a relationship with someone who has been treated for a mental illness]				
120 (15%)	231 (28.9%)	311 (38.9%)	137 (17.1%)	1.58 (0.94)
[I think that most people feel uneasy when someone who has been treated for a mental illness move into the neighbourhood]				
137 (17.1%)	218 (27.3%)	317 (39.7%)	127 (15.9%)	1.54 (0.95)
**Total score/27**				14.44 (5.98)

### Associations between sociodemographics and scores of satisfaction and stigma

Associations between sociodemographic factors and total satisfaction and stigma perception scores are shown in Table 4. A stratified analysis comparing participants from Lower Egypt with those from other regions demonstrated a statistically significant difference in mean satisfaction scores (66.33 ± 17.3 vs. 70.87 ± 18.7, respectively; p = 0.001). Students from regions outside Lower Egypt reported higher satisfaction with telepsychiatry, with a mean difference of 4.54 points (95% CI: 1.84–7.25). No significant regional differences were observed in stigma scores (p = 0.53). STIG-9 stigma scores were significantly higher among students with personal mental health experience (mean = 15.12 ± 6.17) vs those without (13.89 ± 5.62; p = 0.01), and among those with behavioral disorders (16.73 ± 4.77) vs other disorders. The experience of mental illness was also affected significantly by gender (*p* = 0.02), region (*p* < 0.001), field of study (*p* = 0.04), academic level (*p* = 0.001), and economic status (*p* = 0.01) (Table 5). Satisfaction scores were positively correlated with stigma perceptions (*r* = 0.15, *p* < 0.001).

**Table 4:** Associations between sociodemographic factors and total scores of satisfaction and stigma perceptions.

	Satisfaction score			STIG-9 score		
	Mean	SD	*p* value	Mean	SD	*p* value
Gender			0.06			0.91
Male	71.48	18.48		14.94	6.03	
Female	66.99	18.61		15.19	6.23	
Region			0.001*			0.53
Lower Egypt (Delta)	66.33	17.3		14.52	5.8	
Other regions	70.87	18.7		14.23	6.35	
Field of study			0.31			0.09
Medicine and surgery	67.13	18.01		15.23	6.07	
Pharmacy	68.73	14.6		15.07	6.09	
Nursing	76.91	22.59		12.36	4.65	
Other	72.61	21.99		15.29	7.13	
Academic level			0.59			0.99
Preclinical	67.32	19.71		14.96	6.23	
Clinical	68.72	17.94		15.69	6.21	
Intern	69.15	17.91		14.67	6.04	
Economic status			0.14			0.82
High	72	19.14		13.8	6.39	
Middle	68.04	18.56		15.26	6.08	
Low	65.11	19.27		15.33	7.11	
Residence			0.38			0.32
Urban	68.6	18.27		15.51	6.5	
Rural	67.73	19.27		14.5	5.59	
Nationality			0.35			0.26
Egyptian	68.53	17.25		14.56	5.83	
Non-Egyptian	65.23	20.01		13.96	6.50	
Have you ever experienced mental health issues?			0.59			0.01*
Yes	68.25	18.65		15.12	6.17	
No	67.58	17.44		14.02	5.83	
If yes, please specify (N = 303)			0.07			0.04*
Mood disorders (e.g., Depression, Mania, Bipolar)	71.1	19.23		14.67	6.46	
Psychotic disorders (e.g., Schizophrenia)	64.29	10.8		13.29	8.14	
Anxiety disorders	67.64	17.67		15.93	5.71	
Personality disorders	71.8	19.45		14.4	7	
Behavioral disorders (e.g., ADHD, Autism)	66.67	16.1		16.73	4.77	
Others	59.34	20.49		13.55	6.33	

*Significant.

**Table 5:** Association between sociodemographic factors and having mental health issues (n = 799).

	Have you ever experienced mental health issues?				*p* value
	Yes		No		
	N	%	N	%	
Gender					0.02*
Male	85	28.1%	180	36.3%	
Female	218	71.9%	316	63.7%	
Region					< 0.001*
Lower Egypt (Delta)	182	60.1%	355	71.6%	
Other regions	121	39.9%	141	28.4%	
Nationality					0.81
Egyptian	241	79.5%	391	78.8%	
Non-Egyptian	62	20.5%	105	21.2%	
Field of study					0.04*
Medicine and Surgery	239	78.9%	425	85.7%	
Pharmacy	15	5.0%	21	4.2%	
Nursing	11	3.6%	17	3.4%	
Other	38	12.5%	33	6.7%	
Academic level					0.001*
Preclinical	126	41.6%	252	50.8%	
Clinical	98	32.3%	167	33.7%	
Intern	79	26.1%	77	15.5%	
Economic status					0.01*
High	29	9.6%	26	5.2%	
Middle	256	84.5%	453	91.3%	
Low	18	5.9%	17	3.4%	
Residence					0.27
Urban	183	60.4%	280	56.5%	
Rural	120	39.6%	216	43.5%	

* Significant.

% calculated by column.

Tables 6 and 7 present the results of the multivariable linear regression analyses, with no evidence of multicollinearity. Variables independently associated with higher satisfaction scores included region, nationality, and stigma score. Variables independently associated with stigma perceptions included prior experience of mental health issues and satisfaction score.

**Table 6:** Linear regression analysis of variables independently associated with telepsychiatry satisfaction (n = 799).

Variables	Unstandardized coefficients		Sig	95.0% Confidence interval for B		VIF
	B	Std. error		Lower bound	Upper bound	
Age (years)	−0.367	0.287	0.202	−0.930	0.197	1.038
Gender (ref: females)	1.732	1.364	0.204	−0.944	4.409	1.076
Economic status (ref: high)	−1.952	1.888	0.301	−5.658	1.753	1.042
Residence (ref: urban)	−0.132	1.387	0.924	−2.855	2.591	1.223
Nationality (ref: non-Egyptian)	4.506	1.649	0.006*	1.270	7.743	1.173
Have you ever experienced mental health issues? (ref: no)	−0.396	1.310	0.762	−2.968	2.175	1.055
Stigma score	0.454	0.105	< 0.001*	0.248	0.660	1.024
Region (ref: lower Egypt (delta))	5.022	1.509	< 0.001*	2.061	7.984	1.309
Field of the study						
Pharmacy vs medicine and surgery	2.645	3.037	0.384	−3.317	8.606	1.036
Nursing vs medicine and surgery	3.743	3.411	0.273	−2.952	10.438	1.027
Other vs medicine and surgery	2.804	2.239	0.211	−1.591	7.200	1.060

*Significant, VIF = Variance Inflation Factor, B: Unstandardized Coefficients B. R: 0.240; R^2^: 0.058; Adjusted R^2^: 0.044. Note: Regression coefficients represent cross-sectional associations observed within the study sample and should not be interpreted as evidence of causal relationships or temporal effects.

**Table 7:** Linear regression analysis of variables independently associated with stigma perceptions (n = 799).

Variables	Unstandardized coefficients		Sig	95% Confidence interval for B		
	B	Std. error		Lower bound	Upper bound	VIF
Age (years)	−0.099	0.097	0.305	−0.289	0.090	1.038
Gender (ref: females)	0.034	0.459	0.940	−0.867	0.936	1.078
Economic status (ref: high)	0.329	0.635	0.604	−0.918	1.577	1.043
Residence (ref: urban)	−0.566	0.466	0.225	−1.481	0.350	1.221
Nationality (ref: non-Egyptian)	0.333	0.557	0.550	−0.761	1.427	1.184
Have you ever experienced mental health issues? (ref: no)	1.260	0.439	0.004*	0.399	2.120	1.044
Satisfaction score	0.051	0.012	< 0.001*	0.028	0.075	1.036
Region (ref: lower Egypt (delta))	−0.538	0.511	0.293	−1.540	0.465	1.326
Field of the study						
Pharmacy vs medicine and surgery	−1.724	1.020	0.091	−3.727	0.279	1.033
Nursing vs medicine and surgery	−2.026	1.146	0.078	−4.276	0.224	1.024
Other vs medicine and surgery	−1.322	0.753	0.079	−2.799	0.156	1.058

R: 0.215; R2: 0.046; Adjusted R2: 0.033.

Note: Regression coefficients represent cross-sectional associations observed within the study sample and should not be interpreted as evidence of causal relationships or temporal effects.

## Discussion

This study investigated telepsychiatry satisfaction and perceived mental health stigma among a substantial cohort of Egyptian health sciences students with prior service experience. The analysis in this cohort yielded three primary findings: (1) overall satisfaction was moderate, with high ratings for professional courtesy and confidentiality; (2) perceived public stigma remains a significant factor influencing mental health service utilization; and (3) specific sociodemographic variables were independently associated with both outcomes.

The moderate satisfaction levels observed in this study corroborate international literature concerning telepsychiatry acceptance among university populations^17,24^. Specifically, the high scores for the “safety” dimension indicate a robust perception of confidentiality and respectful treatment during virtual encounters. This aligns with evidence suggesting that patients often find the disclosure of sensitive information less stigmatizing and more manageable in a remote context^25,26^. Such perceived privacy is vital in conservative societies where mental health stigma remains pervasive^27^.

Conversely, lower ratings in the access and timeliness dimensions underscore the persistent challenges posed by technological infrastructure and intermittent internet connectivity common in developing nations^28^. Our findings echo previous research in Egypt, which identified that while telemedicine is viewed as inherently beneficial, systemic barriers such as low digital literacy and inadequate resources impede its broader uptake^28^. Furthermore, these results reflect regional trends, such as in Saudi Arabia, where high satisfaction in safety dimensions often contrasts with structural hurdles^29,30^.

Despite their medical background, participants demonstrated significant perceived stigma toward individuals with mental illness. This finding aligns with global documentation of persistent stigma among health sciences students^31,32^ and is particularly pronounced in Middle Eastern settings^33^. A novel insight from this study is the "greater awareness phenomenon," where students with personal experience of mental health issues reported significantly higher stigma scores. This seemingly paradoxical finding suggests that individuals with lived experience are more acutely sensitive to societal manifestations of prejudice^34,35^. Furthermore, our data indicate that students with behavioral conditions (e.g., ADHD) face distinct forms of stigmatization, potentially because their symptoms are more frequently misunderstood or trivialized in professional healthcare environments^35^.

The positive correlation identified between satisfaction and stigma perceptions necessitates careful interpretation. While stigma is traditionally conceptualized as a barrier to care, our findings suggest a more nuanced association in the context of telepsychiatry. One possible explanation is that individuals who are more aware of societal stigma may particularly value the privacy, anonymity, and reduced social exposure afforded by remote mental health services. This may lead to higher reported satisfaction among those with greater perceived stigma. However, this explanation is interpretive and should not be considered causal, particularly given the cross-sectional design of the study.

The reported prevalence of mental health issues in this cohort (37.9%) mirrors the global epidemiological distress observed among health sciences students^36,37^. The predominance of anxiety (39.9%) and mood disorders (38.3%) matches established international patterns^38^. Vulnerability was notably higher among females, consistent with research on gender differences in internalizing disorders^39^. Furthermore, the elevated prevalence among interns compared to preclinical students likely reflects the cumulative stressors associated with the transition to clinical practice and direct patient care^38,40^.

### Implications for practice and policy

The results of this study have several important implications for telepsychiatry services for health sciences students in this Egyptian cohort: First, moderate satisfaction indicates that telepsychiatry is indeed acceptable to this population, but there is still room for improvement, particularly in access, timeliness, and effectiveness. Investments in user-friendly platforms, shorter wait times, and clearer communication about telepsychiatry processes may enhance satisfaction. Second, the persistent stigma identified in this study requires further anti-stigma interventions targeting health sciences students. The students will, in the foreseeable future, become healthcare providers; hence, tackling their stigma goes beyond their own well-being to quality healthcare that will be accorded to patients suffering from psychiatric disorders. Third, those were appropriate demographic variables informing specific approaches.

### Study limitations

There are limitations regarding the interpretation of findings. This study employed convenience sampling through messaging apps, resulting in overrepresentation of Lower Egypt and Medicine/Surgery students, with very small subgroups (Suez Canal/Frontiers n = 8). The respondents were primarily medicine and surgery students from a specific institution, which may limit generalizability to health sciences students in different regions of Egypt. Also overrepresented in the sample is Lower Egypt at 67.2%, while the remaining regions are underrepresented; this may affect the reliability of subgroup analyses for regional comparisons. Response rates per university/governorate were not tracked due to decentralized recruitment by student representatives, precluding representativeness assessments. Due to the small sample sizes across several geographic categories, inferential analyses involving geographic location were restricted to a dichotomized regional variable (Lower Egypt versus other regions) to improve statistical stability and reduce unreliable estimates from sparse subgroups. Therefore, regional findings should be interpreted cautiously, and future studies using more balanced sampling strategies are warranted to explore geographic differences in greater detail. This selection bias may limit generalizability to all health sciences students in Egypt or the MENA region and risks Type I errors in subgroup analyses (e.g., regional satisfaction differences), which justified the decision to avoid multi-region inferential analyses and instead use a dichotomized regional variable in regression models. The cross-sectional component does not allow causal inferences about the relationships among satisfaction and stigma. These patterns should therefore be clarified longitudinally by examining how these constructs change over time and how actual telepsychiatry utilization varies. Findings reflect self-reported experiences toward telepsychiatry, STIG-9 stigma, and mental health care-seeking. While capturing authentic participant perspectives, results remain subject to recall bias, social desirability bias, and reporting inaccuracies inherent to self-report methodologies. Students may have given socially acceptable answers rather than their true views, which in turn could result in underestimates of stigma. Therefore, future studies should consider investigating additional variables, such as technology literacy, past help-seeking behavior in mental health, some individual personality factors, and specific characteristics of the health sciences school environment. Since only a single measure of stigma was used (STIG-9) to assess this phenomenon, it may inadequately represent the multidimensional aspects of mental health-related stigma. It would be more comprehensive if future studies used multiple measures of stigma. Participants were self-reported telepsychiatry users, but no telepsychiatry sessions were conducted as part of this study. Therefore, the findings didn’t reflect observed patient outcomes.

### Future research directions

This study offers insight for future exploration. Longitudinal studies that trace actual changes in satisfaction and stigma resulting from real-world use of telepsychiatry would be quite enlightening about how these constructs evolve with experience. Qualitative work investigating how different health sciences students understand the tele-psychiatry barriers and facilitators would enrich the already well-quantified findings. Comparative studies using probability sampling methods at different Egyptian health sciences schools and, indeed, across MENA countries would expand generalizability and recognize context-specific factors. There is a requirement for studies into the specific telepsychiatry modality, understanding how video compares with telephone and text-based models in respect of patient satisfaction and outcome differential. The perspectives of the mental health providers delivering telepsychiatry to this pool would also be important to understanding the eventual outcome. Finally, initiating intervention studies to evaluate the efficacy of stigma reduction. Recent study among Arab populations have validated culturally adapted instruments for assessing mental illness stigma, such as the Saudi MIAS^41^. However, the present study used the STIG-9 scale because of its brevity, prior validation across multiple populations, and suitability for large online surveys.

## Conclusion

This study highlights the specific experiences of a cohort of Egyptian health sciences students, demonstrating that telepsychiatry may represent an acceptable and feasible modality within this specific population. While satisfaction levels were promising, particularly regarding confidentiality and professional respect, the findings underscore that institutional and socio-cultural barriers—most notably the complex role of perceived stigma and infrastructural limitations—remain important considerations. The identified correlation between service satisfaction and stigma awareness suggests that future digital mental health strategies must be dual-faceted: prioritizing technological reliability while also addressing the nuanced role of stigma in shaping service preferences and experiences. Ultimately, these insights provide a research-validated starting point for policymakers to design more accessible, culturally sensitive, and student-centered telepsychiatry frameworks in Egypt.

## Supplementary Information

Supplementary Information.

## Data Availability

Anonymized dataset available from the corresponding author upon reasonable request. Due to ethical restrictions protecting participant privacy, a full public deposition is not permitted.
