# Supplementary material for: Satisfaction with telepsychiatry and mental health stigma among health sciences students in Egyptian universities
Source: Sci Rep. 2026 Jul 10;16:21609. doi: 10.1038/s41598-026-60516-8 (PMC13354561; doi:10.1038/s41598-026-60516-8)
Supplement: Supplementary file 1 — Supplementary Information. [file 41598_2026_60516_MOESM1_ESM.docx]

Thank you for participating in this study. This survey aims to understand your satisfaction with telepsychiatry (mental health services via video, phone, or online platforms) and stigma around mental health care among college students. Your responses are anonymous and will take approximately 10 minutes to complete.

**Section 1: demographic information**:

1. Age: -

2. Gender: -

-male

-female

3. Academic level: -

-preclinical

-clinical

- Interns

4. Economic status:

- High

-middle

-low

5. Residence: -

-urban

-rural

6. Governorate: -

7- nationality: -

- Egyptian

- Non-Egyptian

**8- Have you ever experienced mental health issues? Please specify………….**

**9- Have you ever used telepsychiatry services?**

**Section 2. Telepsychiatry Patient Satisfaction and Experience Questionnaire**

Answer the following based on your experience with telepsychiatry. **Use a 5-point scale: 1 = Strongly Disagree, 5 = Strongly Agree.**

1. I am satisfied with the length of time I had to wait between my referral and the Telepsychiatry appointment.

2. It was easy to book my Telepsychiatry appointment.

3. During my Telepsychiatry appointment, I was able to see the psychiatrist clearly.

4. During my Telepsychiatry appointment, I was able to hear the psychiatrist clearly.

5. I am confident that the psychiatrist and my health care providers are working as a team.

6. I feel that there was an adequate amount of time allotted for the Telepsychiatry appointment.

7. I felt comfortable during my Telepsychiatry appointment.

8. I believe Telepsychiatry is just as effective as an in-person psychiatry appointment.

9. I was able to get an appointment through Telepsychiatry sooner than an in-person psychiatry appointment.

10. I felt that confidentiality was protected throughout my Telepsychiatry appointment.

11. The psychiatrist understood my concerns.

12. The psychiatrist treated me with courtesy and respect.

13. The psychiatrist explained my diagnosis in a way that I could understand.

14. The psychiatrist involved me in decisions about my treatment plan.

15. The psychiatrist explained the benefits and risks of any medications he/she recommended.

16. I am confident that I will be able to follow the psychiatrist’s recommendations.

17. I understand what to do if I have a mental health emergency following this appointment.

18. The physical location of my Telepsychiatry appointment was convenient for me to get to.

19. I experienced a significant improvement in my mental health while I was waiting for my Telepsychiatry appointment.

20. I experienced a significant decline in my mental health while I was waiting for my Telepsychiatry appointment.

21. Overall, I am satisfied with the Telepsychiatry appointment.

**Section 3: Telepsychiatry and Mental Health Stigma**

| I think that most people... | disagree | somewhat disagree | somewhat agree | agree |
| --- | --- | --- | --- | --- |
| ... take the opinion of someone who has been treated for a mental illness less seriously. | ☐ | ☐ | ☐ | ☐ |
| ... consider someone who has been treated for a mental illness to be dangerous. | ☐ | ☐ | ☐ | ☐ |
| ... hesitate to do business with someone who has been treated for a mental illness. | ☐ | ☐ | ☐ | ☐ |
| ... think badly of someone who has been treated for a mental illness. | ☐ | ☐ | ☐ | ☐ |
| ... consider mental illness to be a sign of personal weakness. | ☐ | ☐ | ☐ | ☐ |
| ... hesitate to entrust their child with someone who has been treated for a mental illness. | ☐ | ☐ | ☐ | ☐ |
| ... do not even take a look at an application from someone who has been treated for a mental illness. | ☐ | ☐ | ☐ | ☐ |
| ... do not enter into a relationship with someone who has been treated for a mental illness. | ☐ | ☐ | ☐ | ☐ |
| ... feel uneasy when someone who has been treated for a mental illness moves into the neighbourhood. | ☐ | ☐ | ☐ | ☐ |

Stigma-9 Questionnaire (STIG-9)

**Table S1: detailed comparisons across all geographic regions**

| **Region** | **N** | **%** |
| --- | --- | --- |
| **Greater Cairo** | 181 | 22.7% |
| **Lower Egypt (Delta)** | 537 | 67.2% |
| **Suez Canal and frontiers** | 8 | 1% |
| **Upper Egypt** | 25 | 3.1% |
| **Alexandria** | 48 | 6% |

**Table S2: full distribution of responses to the telepsychiatry satisfaction questionnaire using a 5-point Likert scale.**

| **Strongly disagree**  **N (%)** | **Disagree**  **N (%)** | **Neutral**  **N (%)** | **Agree**  **N (%)** | **Strongly agree**  **N (%)** | **Mean (SD)** |
| --- | --- | --- | --- | --- | --- |
| **Factor 1: Access and timeliness / 20** | | | | | 12.36 (3.6) |
| 1. I am satisfied with the length of time I had to wait between my referral and the Telepsychiatry appointment. | | | | | |
| 108 (13.5%) | 123 (15.4%) | 374 (46.8%) | 94 (11.8%) | 100 (12.5%) |  |
| 2. It was easy to book my Telepsychiatry appointment | | | | | |
| 120 (15%) | 118 (14.8%) | 301 (37.7%) | 140 (17.5%) | 120 (15%) |  |
| 16. I am confident that I will be able to follow the psychiatrist's recommendations. | | | | | |
| 62 (7.8%) | 97 (12.1%) | 293 (36.7%) | 206 (25.8%) | 141 (17.6%) |  |
| 18. The physical location of my Telepsychiatry appointment was convenient for me to get to. | | | | | |
| 104 (13%) | 122 (15.3%) | 309 (38.7%) | 152 (19%) | 112 (14%) |  |
| **Factor 2: Appropriateness / 15** | | | | | 9.87 (2.85) |
| 8. I believe Telepsychiatry is just as effective as an in-person psychiatry appointment. | | | | | |
| 85 (10.6%) | 137 (17.1%) | 322 (40.3%) | 156 (19.5%) | 99 (12.4%) |  |
| 11. The psychiatrist understood my concerns. | | | | | |
| 65 (8.1%) | 84 (10.5%) | 253 (31.7%) | 220 (27.5%) | 177 (22.2%) |  |
| 14. The psychiatrist involved me in decisions about my treatment plan. | | | | | |
| 72 (9%) | 91 (11.4%) | 266 (33.3%) | 217 (27.2%) | 153 (19.1%) |  |
| **Factor 3: Effectiveness /30** | | | | | 19.12 (5.2) |
| 3. During my Telepsychiatry appointment, I was able to see the psychiatrist clearly | | | | | |
| 85 (10.6%) | 94 (11.8%) | 317 (39.7%) | 153 (19.1%) | 150 (18.8%) |  |
| 4. During my Telepsychiatry appointment, I was able to hear the psychiatrist clearly | | | | | |
| 86 (10.8%) | 100 (12.5%) | 284 (35.5%) | 169 (21.2%) | 160 (20%) |  |
| 5. I am confident that the psychiatrist and my health care providers are working as a team | | | | | |
| 74 (9.3%) | 98 (12.3%) | 280 (35%) | 175 (21.9%) | 172 (21.5%) |  |
| 13. The psychiatrist explained my diagnosis in a way that I could understand. | | | | | |
| 70 (8.8%) | 73 (9.1%) | 262 (32.8%) | 203 (25.4%) | 191 (23.9%) |  |
| 19. I experienced a significant improvement in my mental health while I was waiting for my Telepsychiatry appointment. | | | | | |
| 91 (11.4%) | 104 (13%) | 323 (40.4%) | 168 (21%) | 113 (14.1%) |  |
| 20. I experienced a significant decline in my mental health while I was waiting for my Telepsychiatry appointment. | | | | | |
| 174 (21.8%) | 161 (20.2%) | 293 (36.7%) | 101 (12.6%) | 70 (8.8%) |  |
| **Factor 4: Efficiency /10** | | | | | 6.36 (1.98) |
| 6. I feel that there was an adequate amount of time allotted for the Telepsychiatry appointment. | | | | | |
| 75 (9.4%) | 91 (11.4%) | 344 (43.1%) | 180 (22.5%) | 109 (13.6%) |  |
| 9. I was able to get an appointment through Telepsychiatry sooner than an in-person psychiatry appointment. | | | | | |
| 87 (10.9%) | 115 (14.4%) | 308 (38.5%) | 159 (19.9%) | 130 (16.3%) |  |
| **Factor 5: Safety /25** | | | | | 16.78 (4.87) |
| 7. I felt comfortable during my Telepsychiatry appointment. | | | | | |
| 77 (9.6%) | 103 (12.9%) | 292 (36.5%) | 178 (22.3%) | 149 (18.6%) |  |
| 10. I felt that confidentiality was protected throughout my Telepsychiatry appointment. | | | | | |
| 70 (8.8%) | 105 (13.1%) | 295 (36.9%) | 182 (22.8%) | 147 (18.4%) |  |
| 12. The psychiatrist treated me with courtesy and respect. | | | | | |
| 65 (8.1%) | 81 (10.1%) | 214 (26.8%) | 199 (24.9%) | 240 (30%) |  |
| 15. The psychiatrist explained the benefits and risks of any medications he/she recommended. | | | | | |
| 79 (9.9%) | 79 (9.9%) | 291 (36.4%) | 196 (24.5%) | 154 (19.3%) |  |
| 17. I understand what to do if I have a mental health emergency following this appointment. | | | | | |
| 80 (10%) | 91 (11.4%) | 294 (36.8%) | 184 (23%) | 150 (18.8%) |  |
| **Overall satisfaction** | | | | | |
| 21. Overall, I am satisfied with the Telepsychiatry appointment. | | | | | |
| 67 (8.4%) | 84 (10.5%) | 309 (38.7%) | 192 (24%) | 147 (18.4%) |  |
| **Total score /105** | | | | | 67.84 (17.9) |
